# Dr. C. V. Mehendale

**Published:** 2008

**Authors:** A. Gopalkrishna

**Affiliations:** Consultant Plastic Surgeon, Professor and Head of Department Plastic Surgery, Deccan College of Medical Sciences and allied Hospitals, Hyderabad, India. E-mail: agkga@rediffmail.com

Dr. C.V. Mehendale passed his MBBS in 1950, and M.S. in General Surgery in 1954 from the Seth GS Medical College and King Edward VIIth Memorial Hospital in Mumbai. He then did B.Sc. Anatomy in 1955 and FRCS in 1961. He had worked in different capacities in KEM Hospital, Bombay and LTMG (Sion) Hospital between 1950 and 1955 in the departments of General surgery, Neuro surgery, Thoracic surgery, Experimental surgery, and ENT. Between 1956 and 1964 Dr. Mehendale worked in Frenchay Hospital, Bristol, UK, in the Dept. of Plastic and Jaw Surgery with Mr. Bodenham who was his teacher and mentor. He was Hon. Assistant Professor in Plastic Surgery at the KEM Hospital, Bombay from 1966 to 1976 and was Hon. Associate Professor in Plastic Surgery from 1976 to 1986.

**Figure:**
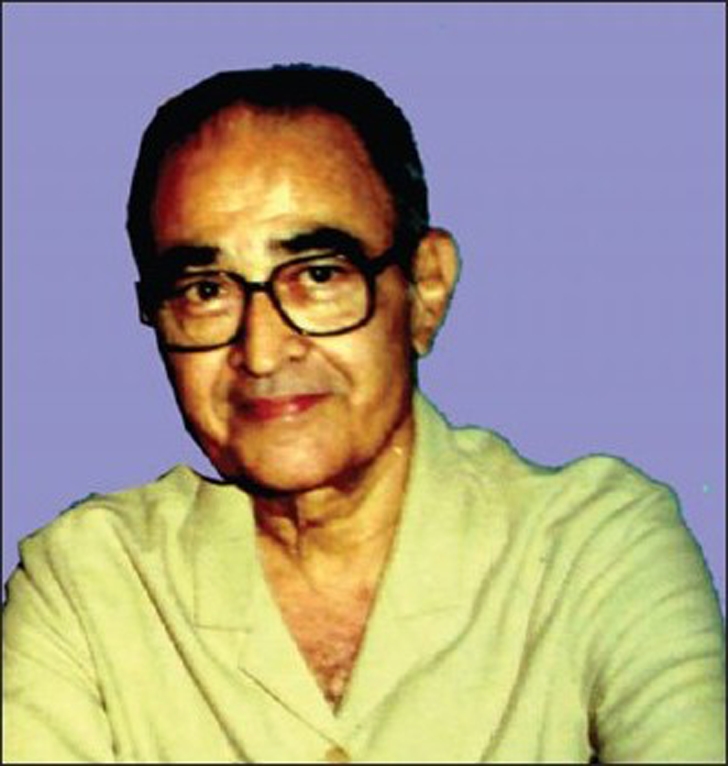
**Dr. Chintamani V. Mehendale** July 27, 1928 to June 21, 2008

He was fondly referred to as “Sir C.V.” by his colleagues, probably because of his deportment, his long years in England and his trademark pipe. He was called “M” by his juniors. He was feared initially by all his juniors, because of his demanding nature. What he expected from the residents was total honesty, truthfulness, and a deep regard for the patients. When they got to know him better, his juniors were totally devoted to him.

In his book “The patient came first,” he believed in total transparency about the treatment, the surgical plan and the possible outcome as far as the patient was concerned. In case of postponement of surgery or alteration in plan he believed in keeping the patient fully informed and he expected no less from his residents.

He was an innovator *par excellence*. In those days of poor availability of both, funds and technology, his innovative skills proved extremely useful to the patients under his care. One of the most impressive things about Dr. Mehendale was his quest for knowledge. He could not use a new technology or a new instrument without going into the basic science behind it. Only after a clear understanding of the scientific basis did he use any technology and he encouraged his students to do the same. Even in objects of daily use, which others might use without a second thought, he would go into the basic scientific principles.

Dr. Mehendale embodied all the great qualities expected in a plastic surgeon. He had a thorough knowledge of the basic subject. He made a meticulous plan for every surgery but did not mind deviating from the plan when necessary, which was seldom. He insisted on always using the correct instrument for the right job. He has even invented a few instruments which he never patented. Notable among these are, a pen holding grip needle holder for cleft palate (A similar needle holder is now being used for Micro Surgery), a hook for elevation of the zygoma with variable grip, and what is very commonly used by a lot of us - the wire twister. He is said to have invented a book stand for reading a book while lying down on bed, a head lamp to illuminate a small part of a page for concentrated reading at exam time and an elaborate system of electrical wires to electrocute a crow that was disturbing him while he was preparing for his MS exams!

Dr. C.V. Mehendale was an avid photographer, so much so that he had his own dark room facility at home. He even assembled a system to create double-flash illumination on a subject with a specific time gap between the flashes to get a specific effect. His interest in the Indian classical music and his knowledge of the physics of sound production was unique and manifested itself in developing “Anil Vadyam” a new musical instrument which was presented at the Bombay music college by Dr. Mehendale himself playing raga jayajayawanthi.

He was deeply concerned when a citizen's action threatened someone's life, particularly that of a child. It was not very uncommon for him to stop his car and instruct a mother to walk with the child held away from the side of the vehicular traffic. Disregard for the safety of children agitated him. Such was the soft side of a tough looking personality.

In the words of Dr. Mehendale himself “there are surgeons who do plastic surgery and then there are plastic surgeons”.

Dr. C.V. Mehendale was a plastic surgeon in the true mould.

